# Beta-Cell Function and Its Underlying Mechanism 2015

**DOI:** 10.1155/2016/7638120

**Published:** 2015-12-13

**Authors:** Yanbing Li, Chen Wang, Bilal Omar, Li Chen

**Affiliations:** ^1^Department of Endocrinology, The First Affiliated Hospital, Sun Yat-sen University, Guangzhou 510080, China; ^2^Department of Endocrinology and Metabolism, Shanghai Jiao Tong University Affiliated Sixth People's Hospital, Shanghai 200233, China; ^3^Department of Clinical Sciences, Biomedical Center, Lund University, 22184 Lund, Sweden; ^4^Department of Endocrinology, Qilu Hospital of Shandong University, Jinan 250012, China

Dynamic changes in beta-cell function during the development and progression of diabetes mellitus are well characterized. However, the driving force and underlying mechanism are poorly understood. In this special issue, we selected multiple original articles and one review which are aiming to explore the beta-cell function and its underlying mechanism from clinical and basic research aspects.

X. Hou et al. investigated *β*-cell function in 818 newly diagnosed drug naive type 2 diabetic patients grouped by HbA1c values (≤6.5%, 6.5–7%, 7-8%, 8-9%, and ≥9%). They showed that individuals with HbA1c of 6.5–7% exhibited increased HOMA-*β* as compared to those ≤6.5%. However, the indices decreased substantially with further increment of HbA1c levels. Betatrophin and irisin are two recently identified hormones which may participate in regulating pancreatic *β*-cell function. L. Wang et al. compared the two hormone levels in 20 NGT and 120 type 2 diabetic patients. Though betatrophin was found significantly elevated, while irisin levels significantly decreased in patients with type 2 diabetes, partial correlation analysis failed to show a correlation between either betatrophin or irisin level and those of *β*-cell function-related variables.

Particular experience using short-term intensive insulin treatment as the initial management in newly diagnosed type 2 diabetic patients was shared in this issue. J. Ma et al. used intravenous insulin infusion prior to subcutaneous insulin pump therapy in 65 type 2 diabetic patients with HbA1c ≥ 11.80%. They showed that total daily insulin dose in pump therapy was determined by the change of blood glucose in response to intravenous insulin infusion. In another paper, W. Ke et al. randomized 39 newly diagnosed type 2 diabetic patients into either short-term CSII alone or in combination with liraglutide treatment for 12 weeks. They showed that patients with combination therapy achieved euglycemia in shorter time, and their increment of AIR was significantly higher. However, after stopping liraglutide, its effect on beta-cell function disappeared completely.

The basic research papers are interesting. F. Li et al. isolated ISCs from islets of Goto-Kakizaki rats, determined the gene profiles, and assessed their effects on beta-cell function and survival. They concluded that ISCs presented in fibrotic islet of GK rats might be special PSCs, which impaired beta-cell function and proliferation and increased beta-cell apoptosis. Islet brain 1 (IB1) is a candidate gene for diabetes that is required for beta-cell survival and glucose-induced insulin secretion (GSIS). S. Brajkovic et al. showed that chronic exposure of MIN6 cells and isolated rat islets cells to palmitate led to reduction of IB1 mRNA and protein content. Suppression of IB1 level mimicked the harmful effects of palmitate on the beta-cell survival and GSIS. Conversely, ectopic expression of IB1 counteracted the deleterious effects of palmitate on the beta-cell survival and insulin secretion. The lipid droplet-associated proteins FSP27/CIDEC and LSDP5 are associated with hepatic steatosis and insulin sensitivity. Y. Zhu et al. showed that fenofibrate treatment decreased hepatic triglyceride content and FSP27/CIDEC protein expression in mice fed with an HF diet. In contrast, LSDP5 was highly expressed in humans, with elevated expression observed in the fatty liver.

In a review article, X. Luo et al. reviewed the roles of pyruvate, NADH, and complex I in insulin secretion. They proposed that complex I played a crucial role in the pathogenesis of *β* cell dysfunction in the diabetic pancreas, based on the fact that chronic hyperglycemia overloads complex I with NADH and leads to enhanced complex I production of reactive oxygen species (ROS), which have been implicated in the pathogenesis of diabetic hyperglycemia.

The articles in this issue bring about some new perspectives that add to our knowledge about beta-cell function and its underlying mechanism. We hope that our readers will be enjoying them.

*Yanbing Li* *Yanbing Li* *Chen Wang* *Chen Wang* *Bilal Omar* *Bilal Omar* *Li Chen* *Li Chen*

